# Hints on the Medicinal Effect of Living with Cows, in Pulmonary Consumption

**Published:** 1800-01

**Authors:** W. Blair

**Affiliations:** Surgeon of the Lock Hospital and Finsbury Dispensary, etc.


### *Hints on the Medicinal Effect of Living with Cows, in Pulmonary Confumption. Communicated by* Mr. Blair, Surgeon of the Lock Hofpital and Finfbury Difpenfary, &c.

---

### *To the Editors of the Medical and Phyfical Journal.*

Gentlemen,

We are faid to have the felicity of living in an age "diftinguifhed from all the preceding, by great practical difcoveries immediately refulting from fpeculation;" and in which the public are "too intelligent to be duped by thofe artifices that have fo often given Medical Men vogue in the great world." A gentleman who, by his *aerial* fpeculations and difcoveries, is exalted far above his profeffional brethren, feems of opinion, that the propofal "*of living with cows*" has been treated too faftidioufly by practitioners in general. "As nobody, to my knowledge," he tells us, "has been moved by my exhortations (in Confiderations on Airs, in the Weft Country Contributions, and in my Effay on Confumptions,) to try the effect of living with cows in phthifis pulmonalis, I have lately put this practice to the teft of experiment." Perhaps one of the reafons why this treatment meets with but little regard, may be, that it has not fo much of the fafcinating merit of novelty to recommend it, as your correfpondent fuppofes. I have made no inquiries among my medical friends on this fubject; but, fo far as my own recollection ferves me, I think the experiment has been often and long ago tried, with no great fuccefs.

I beg leave to tranfcribe a few lines from a work lying before me, in which the practice here alluded to is explicitly mentioned. By taking the trouble to look over different medical writings, for that exprefs purpofe, other notices of the fame

ſame kind might probably be found. "*Paucis abhinc annis ſtabula vaccarum habitare phthiſici jubebantur, at ſiniſtro prorſus eventu. Murray feminam vidit, fere ſuffocatam, etſi unica in bubili vacca ſtabularetur.*" (JOSEPHI QUARIN, Animadverſiones practicæ in diverſos morbos, cap. v. p. 102, 8vo. Viennæ, 1786.) I will not conjecture what motive induced practitioners to relinquiſh ſo curious a project; but the ſcheme of aſſociating with cows, I imagine, would accord leſs with the dignity and accompliſhed habits of Engliſh phthiſical ladies, than another plan of cure ſuggeſted by the ſame intelligent gentleman, viz. that of procuring a comfortable retreat among the poliſhed natives of Egypt.

I remain, GENTLEMEN, &c.

*Great Ruſſel Street, Bloomſbury,*

*Dec.* 16, 1799. W. BLAIR.

